# Accidental falling in community-dwelling elderly with chronic kidney disease

**DOI:** 10.1007/s11255-018-1992-9

**Published:** 2018-10-15

**Authors:** Namiko A. Goto, Marije E. Hamaker, Hanna C. Willems, Marianne C. Verhaar, Mariëlle H. Emmelot-Vonk

**Affiliations:** 1 Dianet Dialysis Center, Utrecht, The Netherlands; 2 Department of Geriatrics, University Medical Center Utrecht, Heidelberglaan 100, 3584 CX Utrecht, The Netherlands; 3 Department of Geriatrics, Diakonessenhuis Utrecht, Utrecht, The Netherlands; 4 Department of Geriatrics, Academic Medical Center Amsterdam, Amsterdam, The Netherlands; 5 Department of Nephrology and Hypertension, Utrecht University, Utrecht, The Netherlands

**Keywords:** Accidental falls, Aged, Aged 80 and over, Chronic renal insufficiency, Chronic kidney failure

## Abstract

**Purpose:**

The aim of the current study was to evaluate the association between a decreased estimated glomerular filtration rate (eGFR) and accidental falling in elderly patients who visited the day clinic of the department of geriatric medicine of the University of Medical Center Utrecht (UMCU).

**Study design:**

A cross-sectional analysis with people aged ≥ 65 years of the Utrecht Cardiovascular Cohort was performed. Patients were stratified into different stages of kidney disease (< 45, 45–59, and ≥ 60 ml/min per 1.73 m^2^). Logistic regression models were used to evaluate the association between chronic kidney disease and falling.

**Results:**

Our analysis included 1000 participants with a mean age 79.4 (± 6.6) years, of whom 38% had an eGFR of < 60 ml/min per 1.73 m^2^ and 17% < 45 ml/min per 1.73 m^2^. Univariate analysis showed a significant higher prevalence [odds ratio 1.75 (95% confidence interval 1.21–2.53; *p* ≤ 0.01)] of falling in the population with an eGFR < 45 ml/min per 1.73 m^2^ compared to patients with an eGFR ≥ 60 ml/min per 1.73 m^2^. After correcting for multiple potential confounders in the multivariate analysis, this association was no longer present.

**Conclusions:**

In geriatric patients ≥ 65 years, patients with a decreased eGFR fall more often than patients with a preserved kidney function. This seems to be related with the risk profile of patients with CKD and not with a decreased eGFR itself, as after correcting for potential confounders no association remained. Nevertheless, accidental falling is a highly prevalent problem in the elderly CKD population. Therefore, nephrologists should actively ask about accidental falling, and thereby screen for high-risk patients.

## Introduction

Accidental falling is a common problem in the elderly population. Approximately one-third of people aged ≥ 65 years fall each year, increasing to around 40% for those ≥ 70 years of age [1]. These rates are even higher in institutionalized elderly [2]. A fall is defined by the World Health Organization (WHO) as an event which results in a person coming to rest inadvertently on the ground or floor or other lower level [1]. Falls can result in serious injury, loss of independence, functional decline, and death. Unintentional injuries are the fifth leading cause of death in older adults, and falls are responsible for two-thirds of these deaths [3]. Among patients with chronic kidney disease (CKD) who experienced a serious fall incident, nearly one in five died within a year of the fall [4]. Subsequently, falls place a substantial economic burden on society [5]. The majority of falls have a multifactorial etiology. Important biological risk factors are age, gender, physical decline, cognitive impairment, depression, and comorbidity. Important behavioral risk factors are the use of multiple medications, malnutrition, and excess alcohol use [1].

In addition, CKD is a global public health issue and is associated with increasing age [6, 7]. Patients with CKD have a higher rate of cognitive impairment, depressive symptoms, exhaustion, impaired mobility, polypharmacy, and frailty [8]. Patients with CKD are also more vulnerable for fractures, due to renal osteodystrophy [9]. The coexistence of factors such as polypharmacy, comorbidities, cognitive impairment, and depression suggests that patients with different degrees of CKD are more likely to fall than the general population. However, it is not known if this potential effect on falling is the result of this high-risk profile or also a direct result of a decreased estimated glomerular filtration rate (eGFR). Our hypothesis is that patients with a decreased eGFR fall more often as a result of the direct consequences of CKD on metabolic disturbances, such as anemia, low alfacalcidol levels, uremia, and disturbances in electrolytes. It is important to understand whether the risk profile, the decreased eGFR, or both are associated with falls, because it can help to better identify and treat high-risk patients that need fall prevention and thereby prevent morbidity and mortality.

A few studies have focused on the association between falling and end-stage kidney disease (ESKD), mainly in the population with renal replacement therapy (RRT) (hemodialysis, peritoneal dialysis) [10–12]. Falls in elderly patients on RRT are highly prevalent, and associated with severe morbidity risks and high mortality [10–12]. However, a large part of the CKD population will never progress to ESKD and therefore never need RRT. The few studies that assessed the association between falling and CKD found inconsistent findings [4, 13–16]. Two studies showed no association, but these studies focused on recurrent falling in nursing home residents only or on serious fall incidents [4, 16]. Other studies that did show an association were post hoc analyses of medication randomized controlled trials [14, 15]. No cross-sectional or prospective research is available on falling in community-dwelling geriatric subjects with CKD [12].

The aim of the current study was to evaluate the association between a decreased eGFR and accidental falling in elderly patients who visited the day clinic of the department of geriatric medicine of the University of Medical Center Utrecht (UMCU).

## Materials and methods

### Study participants

A cross-sectional study was performed using the Utrecht Cardiovascular Cohort. This is a cohort study conducted at the UMCU. All patients that visited one of the geriatric outpatients clinics at the department of geriatric medicine in the UMCU (memory clinic, falls clinic, and general geriatric day hospital) in the period from January 1, 2011 to December 31, 2014 were included. As part of usual care, all these patients underwent a comprehensive geriatric assessment consisting of a physical examination, cognitive and mobility tests, and laboratory testing. In addition, patients filled out a questionnaire concerning their general health and history of accidental falls. Data of these outpatients were collected in a database by the nurses and physicians working at the day clinic. The study protocol was approved by the Medical Ethics Committee of the UMCU.

For this analysis, patients were excluded if age was under 65 years, if no kidney function was available, when on RRT, or if no questionnaire about accidental falls was available.

### Data collection

The following data were extracted from the database: age, gender, living situation, medical history, intoxications, history of falls, number of medications, comorbidity, postural hypotension, body mass index, physical function, cognition, symptoms of depression, data of functional assessment (Katz-15 scale), and blood test results.

Falls were defined as a fall in the previous year. Frequent falls were defined as two or more falls in the past year. Comorbidity was scored by using the Charlson comorbidity index (CCI) [17]. This index is a widely used score to predict the risk of death from comorbid disease.

Cardiovascular disease was defined as a history of myocardial infarction, transient ischemic attack, stroke, or peripheral artery disease. Postural hypotension was defined as a fall in systolic blood pressure of at least 20 mmHg or diastolic blood pressure of at least 10 mmHg when a person assumes a standing position after lying down for a period of at least 10 min. Blood pressure was measured after 1 and after 3 min after assuming standing position. Physical function was assessed by evaluating if the patient walked with the help of a walking aid. Functional assessment was assessed by the Katz-15 questionnaire [18]. This is a combination of the Katz-6 ADL (assessing basic activities of daily living) and the iADL (nine items assessing everyday functional, or instrumental competence). A higher score indicates higher dependency in basic and instrumental daily living. Number of medications was dichotomized in polypharmacy or not using a cut-off point of ≥ 5 medications.

Cognition was based on the Mini Mental Examination (MMSE) [19]. This is a standardized and valid test with the purpose of screening for cognitive impairment. In this test orientation, attention, calculation, language, immediate and short-term recall, and visual construction are tested. The MMSE score ranges from 0 to 30 points. A higher score indicates better cognitive functioning. Impaired cognition was defined as an MMSE score < 24 or a known diagnosis of mild cognitive impairment or dementia. When no MMSE was performed, because there was no indication, patients were considered to have a normal cognitive function.

Symptoms of depression were measured by the short version of the Geriatric depression Scale (GDS-15) [20]. If a patient had a score >  5, patients were considered depressive.

### Measures of kidney function

Measures of kidney function were calculated by the CKD epidemiology collaboration (CKD-EPI) equation (estimation of glomerular filtration rate based on creatinine, age, and sex). For the equation Caucasian race was assumed. An impaired kidney function was defined as an eGFR < 60 ml/min per 1.73 m^2^. Kidney function was categorized into three groups according to the cut-off values recommended by the US National Kidney Foundation Kidney Disease Outcomes Quality Initiative Guidelines (K/DOQI guidelines); ≥ 60, 45–59, and < 45 ml/min per 1.73 m^2^ [21]. Because of the low number (*n* = 38) of patients with an eGFR < 30 ml/min per 1.73 m^2^, no separate group was created for this kidney function.

### Statistical analysis

Data were analyzed using SPSSS software (IBM SPSS statistics version 21). Because the baseline dataset showed some missing data (all variables < 10% missing), we performed multiple imputation to replace missing data as implemented by SPSS, version 21. This procedure was repeated ten times, resulting in ten different ‘complete’ datasets. The final results were averaged across these datasets and were used for logistic regression. Differences between group results were evaluated using the unpaired *t* test for continuous parametric data, Kruskal–Wallis for non-parametric continuous data, and Chi-square for categorized variables. For differences between continuous parametric data in the different kidney groups, the One-Way Anova was used. Categorical variables were reported as proportions; continuous variables were reported as means with standard deviations or medians with interquartile range for non-parametric data.

To investigate the association between falling and the different stages of a decreased eGFR, logistic regression analysis was performed. Potential risk factors were chosen a priori [22]. Different models with potential confounders were used to calculate the adjusted odds ratio (OR). Model 1 is unadjusted. Model 2 adjusts for age and gender. Model 3 adjusts for model 2 and Katz-15, the use of a walking aid and polypharmacy, model 4 (final model) adjusts for model 3 and cardiovascular disease, GDS, cognitive impairment, postural hypotension, and alcohol use. Adjusted OR were calculated for accidental falling and frequent falling. Because the patients visiting the geriatric outpatients clinic are a very heterogeneous population, additional subgroup analysis was performed for the different clinics (memory clinic, falls clinic, general geriatric day hospital). A two-sided probability of *p* < 0.05 was considered statistically significant. Outcomes were calculated with a 95% confidence interval (95% CI).

## Results

This analysis includes all patients visiting the geriatric day clinic between 2011 and 2014, aged ≥ 65 years (*n* = 1385) who filled out the questionnaire about accidental falls (*n* = 1012). Patients with no available kidney function and patients on RRT were excluded (*n* = 12), leaving 1000 participants for analysis. Patients that were excluded from analysis because of a missing questionnaire about accidental falls are shown in Table 3 in Appendix. In general, these excluded patients were comparable to our study population. Only differences were the need of professional help, more myocardial infarction, and more impaired cognition. The characteristics of the included patients are shown in Table 1. The mean age of the population was 79.4 ± 6.6 years. The mean eGFR was 64.9 ± 19.3 ml/min per 1.73 m^2^. Of the total population, 38% of the patients had an eGFR < 60 ml/min per 1.73 m^2^ of whom 17% had a kidney function < 45 ml/min per 1.73 m^2^. Compared to patients with a normal kidney function, patients in the group < 45 ml/min per 1.73 m^2^ were older (82.2 ± 6.6 vs. 78.0 ± 6.3), used more medication (84.8% vs. 65.6%), and had a higher comorbidity burden with more cardiovascular disease (47.0% vs. 27.7%) and diabetes (34.9% vs. 23.0%). They were also more dependent in ADL and iADL. Moreover, there was a higher use of a walking aid (70.2% vs. 48.8%) and postural hypotension (47.1% vs. 37.2%). Compared to the other day clinics, patients that were referred to the general geriatric day hospital had the highest rate of impaired kidney function (42.9%, 35.5%, and 27.0%, in the 45–59 and ≥ 60 ml/min/1.73 m^2^, respectively, *p* ≤ 0.01). On the other hand, patients that were referred to the memory clinic had a relatively low rate of impaired kidney function (49.4% in the preserved kidney group, 44.4% and 35.7% in the group with eGFR 45–59 and < 45 ml/min/1.73 m^2^, *p* ≤ 0.01). There were no significant differences in gender, smoking status, alcohol use, cognition, and symptoms of depression between the different groups.

**Table 1:** Baseline characteristics

Characteristic	CKD-EPI in ml/min per 1.73 m^2^			*p* value
	< 45 (*n* = 168)	45–59 (*n* = 214)	≥ 60 (*n* = 618)	
Age, mean ± SD	82.2 (6.6)	81.4 (6.2)	78.0 (6.3)	< 0.01
Female, *n* (%)	108 (64.3%)	144 (67.3%)	369 (59.7%)	0.12
Referred to				
Memory clinic	60 (35.7%)	95 (44.4%)	305 (49.4%)	< 0.01
Falls clinic	33 (19.6%)	39 (18.2%)	138 (22.3%)	0.40
Geriatric assessment clinic	72 (42.9%)	76 (35.5%)	167 (27.0%)	< 0.01
CCI, mean ± SD	7.0 (2.5)	5.6 (2.0)	4.8 (1.9)	< 0.01
Diabetes, *n* (%)	58 (34.9%)	60 (28.0%)	142 (23.0%)	0.01
Cardiovascular disease, *n* (%)	79 (47.0%)	77 (36.0%)	171 (27.7%)	< 0.01
Current smoker, *n* (%)	18 (11.6%)	27 (13.2%)	75 (12.8%)	0.90
Alcohol use, *n* (%)	62 (39.2%)	88 (43.3%)	271 (46.0%)	0.30
KATZ-15, median (IQR)	6.0 (6.0)	5.0 (6.0)	4.0 (5.0)	< 0.01
Postural hypotension, *n* (%)	72 (47.1%)	103 (51.2%)	219 (37.2%)	< 0.01
Walking aid, *n* (%)	113 (70.2%)	124 (61.1%)	295 (48.8%)	< 0.01
Polypharmacy, *n* (%)	140 (84.8%)	149 (70.6%)	381 (65.6%)	< 0.01
Cognitive impairment, *n* (%)	64 (38.1%)	82 (38.3%)	200 (32.4%)	0.17
Symptoms of depression, *n* (%)	36 (22.0%)	36 (17.1%)	119 (19.6%)	0.50
Accidental fall, *n* (%)	119 (70.8%)	134 (62.6%)	364 (58.9%)	0.02
Frequent falls, *n* (%)	79 (49.4%)	99 (46.5%)	253 (41.8%)	0.17

The following variables had missing data referred to (1.5%), diabetes (0.2%), current smoker (5.4%), alcohol use (5.0%), KATZ-15 (7.6%), postural hypotension (5.7%), walking aid (3.1%), polypharmacy (4.3%), symptoms of depression (2.0%), frequent falls (2.2%)

*SD* standard deviation, *IQR* interquartile range, *CCI* Charlson comorbidity index, *Accidental fall* fall in the previous year, *Frequent falls* ≥ 2 falls in the previous year

Overall, 617 (61.7%) patients had an accidental fall in the previous year. This rate was the highest in the < 45 ml/min group (70.8%). Frequent falling (≥ 2 falls in the past year) was also more common in the < 45 ml/min group (49.4% vs. 41.8% in the population with a preserved kidney function) but this difference was not significant (*p* = 0.17) (Table 1). Of all patients, 20.9% was referred to the falls clinic. If these patients were excluded from analysis, the fall rate was 54.8% in the main population and 68.3% in the population with an eGFR < 45 ml/min per 1.73 m^2^.

### Impaired kidney function and accidental falling

ORs for the different models analyzing the association between decreased eGFR and falls are shown in Table 2. In the unadjusted model (model 1) an association was found between an eGFR < 45 ml/min and accidental falling (OR 1.70, 95% CI 1.17–2.45). After correcting for potential confounders this association was no longer present (final model OR 1.08, 95% CI 0.72–1.62). A similar result was found for frequent falls: non-adjusted OR 1.43 (95% CI 1.01–2.02) and OR 0.94 (95% CI 0.64–1.38) in the fully adjusted model.

**Table 2:** Association between a decreased eGFR and falls

eGFR-group	Model 1			Model 2			Model 3			Model 4		
	OR	CI (95%)	*p* value	OR	CI (95%)	*p* value	OR	CI (95%)	*p* value	OR	CI (95%)	*p* value
Accidental falls												
0–45 vs. > 60	1.70	1.17–2.45	< 0.01	1.37	0.94–2.02	0.10	1.12	0.75–1.67	0.60	1.08	0.72–1.62	0.72
45–59 vs. ≥ 60	1.17	0.85–1.61	0.34	0.97	0.69–1.35	0.83	0.90	0.64–1.27	0.54	0.87	0.61–1.23	0.42
Frequent falls												
0–45 vs. > 60	1.43	1.01–2.02	0.04	1.27	0.89–1.81	0.20	0.98	0.67–1.44	0.93	0.94	0.64–1.38	0.75
45–59 vs. ≥ 60	1.20	0.88–1.64	0.26	1.07	0.77–1.48	0.67	0.98	0.70–1.38	0.91	0.94	0.67–1.33	0.74

Model 1 unadjusted, model 2: adjusted for age and gender, model 3: adjusted for model 2 Katz-15, the use of a walking aid, polypharmacy. Model 4: adjusted for model 3 + diabetes, cardiovascular disease, alcohol, postural hypotension, cognitive impairment, symptoms of depression

*eGFR* estimated glomerular filtration rate in ml/min/1.72 m^2^, *Accidental fall* fall in the previous year, *Frequent falls* ≥ 2 falls in the previous year, *OR* odds ratio, *CI* (95%) 95% confidence interval

Additional subgroup analyses for the different geriatric outpatients are shown in Tables 5, 6, and 7 in Appendix. An independent association between a decreased eGFR and falling (final model OR 2.08, 95% CI 1.06–4.08) was found in the group of patients visiting the general geriatric day hospital. No association was found for patients visiting the falls or memory clinic. Patients visiting the general geriatric day hospital were more frequently impaired in functional status (median 5.0 vs. 4.0 in both the memory and the fall clinic), had more polypharmacy (78.0% vs. 74.0% in the falls clinic and 62.5% in the memory clinic), had more symptoms of depression (26.0% vs. 17.3% in the memory clinic and 14.4% in the falls clinic), and had more frequently an eGFR < 30 ml/min/1.73 m^2^ (8.9% vs. 3.7% in the memory clinic and 2.4% in the falls clinic). More detailed baseline characteristics for the different outpatient clinics are shown in Table 4 in Appendix.

## Discussion

In this cross-sectional study involving 1000 community-dwelling geriatric subjects aged 65 years and over, the prevalence of accidental falling was high (61,7%). Patients with an eGFR < 45 ml/min per 1.73 m^2^ had significantly higher rates of accidental falling (OR 1.70, 95% CI 1.17–2.45) compared to patients with a preserved kidney function. Patients in this group also fell more frequently (OR 1.43, 95% CI 1.01–2.02) than patients with a preserved kidney function. After correction for potential confounders there was no independent association between a decreased eGFR, accidental falling, and frequency of falling in the overall group. However, in sub-analysis, there was an independent association between an eGFR < 45 ml/min per 1.73 m^2^ and falling in the patients that were referred to the general geriatric day hospital (OR 2.08, 95% CI 1.06–4.08).

The rate of accidental falling in our population is high compared to other studies in the elderly population as well as in dialysis patients. A review about falling in community-dwelling adults reported that 35–45% of persons over 65 years old fall every year, and up to 50% of those over 80 years old [23]. A study performed in an elderly dialysis population found a percentage of 55% [24]. The high frequency of falling in our population is most likely due to the frail and elderly (mean age > 80 years) population visiting the geriatric clinic. Also 1 in 5 patients was specifically referred to the falls clinic. However, if these patients are excluded from analysis, the fall rate remained high at a rate of 54.8% in the main population and 68.3% in the population with an eGFR < 45 ml/min per 1.73 m^2^.

Our results suggests that a decreased eGFR does not make a direct contribution on accidental falling in the overall geriatric population. There are several possible explanations why patients with a decreased eGFR in our study population fall more often. Firstly, CKD (and treatment for optimization of CKD) can lead to risk factors of falling. In our study population, patients with a decreased eGFR were older, used more medication, had a higher comorbidity burden, were more dependent in daily life, had more postural hypotension, and were more immobile. These are all risk factors for falling [1, 22]. Secondly, the main causes of CKD in the elderly population are cardiovascular risk factors, including hypertension and diabetes. These comorbidities are also associated with falling [22, 25]. Therefore, it is likely that patients with a decreased eGFR fall more often because of their risk profile and not by the direct consequences of a decreased eGFR.

Similar to our main finding, previous studies also did not find an association between accidental falling and a decreased eGFR. One large prospective cohort study in the United States in a population with patients of ≥ 65 years found no relationship between a decreased eGFR (< 45, 45–59, and ≥ 60 ml/min per 1.73 m^2^) and serious fall incidents. However, not only the serious fall incidents have consequences. Minor fall incidents can also lead to fear of falling, depression, social isolation, and institutionalization. Patients in this study were also younger, and less frail than in our study population (less cognitive impairment, less depression, and less immobility) [4]. In a retrospective study in nursing home patients with a history of falls, a decreased eGFR was not an (in)dependent risk factor for recurrent falling or injurious falls [16]. The study population was comparable in age and kidney function (35% had a decreased eGFR vs. 38% in our study population), but had a higher use of a walking aid. Two studies that did find an association between a decreased eGFR and falling were sub-analysis of double-blind placebo-controlled trials that studied the influence of calcitriol and estrogen on falling [14, 15]. These studies were performed in a healthier and younger population (severe chronic illness was excluded) and did not correct for most geriatric domains. Neither of these studies assessed the relationship between specifically stage 4 or stage 5 of CKD and accidental falling.

However, in the group that was referred to the general geriatric day hospital, we did find an independent association between a decreased eGFR and accidental falling. Our hypothesis is that this association is a consequence of a higher frequency of a severely impaired kidney function (< 30 ml/min/1.73 m^2^) in the group of patients visiting the general geriatric day hospital. Since large population studies did find an independent association of mild-to-moderate CKD with gait disturbances and incident mobility [26–28], it could be possible that this effect on accidental falling is seen in a more advanced stage of CKD. Unfortunately, we could not perform an analysis on the association between falling and lower levels of eGFR because of the small groups (*n* = 5 in < 15 ml/min per 1.73 m^2^ and *n* = 33 in 15–29 ml/min per 1.73 m^2^). Further research in patients with more severely impaired kidney function is needed to explore this hypothesis.

This study has several limitations. Due to the cross-sectional character of the study no falls incidence could be calculated. Furthermore, data on falls were self-reported by the patients and may be affected by recall bias (especially in patients visiting the memory clinic). As prior research has shown that elderly patients are often unable to recall falls over a longer period of time [29], it is possible that the fall prevalence may be even higher in our study population. There also was no information available on severity of a fall and the etiology of the fall. In our database, we did not specify type, dosage, and duration of medication. Therefore, we could not correct for the effect of fall-associated medication (sedatives, antidepressants). In addition, eGFR is not static and fluctuates over time. In our study population, we only measured eGFR once (independently of moment of fall), and therefore these markers may not reflect true values of kidney function. Furthermore, as levels of creatinine are affected by muscle mass, eGFR can be overestimated in the frail population. Despite these limitations, our study provides a large population of frail elderly with a high rate of accidental falling. Because a geriatric assessment was performed in every patient, much information was available on potential risk factors for falling.

The associations that we report in this study may have implications for the care of elderly patients with CKD. Previous research in the general population showed that less than half of the patients who experienced a fall reported this to a healthcare provider [30]. Therefore, it is very important for the nephrologist (or general practitioner) to ask patients in this population about previous falls as most patients would not mention this problem by themselves. Several studies have also shown that fall prevention measures can significantly reduce the amount of falls and hospitalizations in elderly patients with CKD [31–33]. Therefore in patients with a previous fall, fall prevention should be performed to prevent morbidity and mortality.

In conclusion, patients with a decreased eGFR have more accidental falling and tend to fall more frequently than patients with normal kidney function. This is most likely due to the risk profile of the CKD patient, and not due to the decreased eGFR. Further research should focus on the relationship between lower levels of eGFR and falling and risk factors in the CKD population to improve patient selection.

## Appendix

See Tables 3, 4, 5, 6, and 7.

**Table 3:** Characteristics of patients with missing questionnaires

Characteristic	Questionnaire available (*n* = 1004)	Questionnaire missing (*n* = 366)	*p* value
Age, mean ± SD	79.4 (6.6)	79.7 (7.1)	0.06
Female, *n* (%)	623 (62.1%)	225 (61.5%)	0.85
Living with professional help, *n* (%)	613 (61.2%)	235 (68.1%)	0.02
CCI, mean ± SD	5.3 (2.2)	5.4 (2.1)	0.10
∙ Diabetes, *n* (%)	260 (25.9%)	96 (26.4%)	0.85
∙ CVA, *n* (%)	121 (12.1%)	49 (13.5%)	0.37
∙ TIA, *n* (%)	95 (9.6%)	35 (9.7%)	0.93
∙ Myocardial infarction, *n* (%)	119 (11.9%)	58 (16.0%)	0.05
∙ Peripheral arterial disease, *n* (%)	72 (7.2%)	25 (6.9%)	0.83
Current smoker, *n* (%)	120 (12.6%)	48 (15.5%)	0.19
Alcohol use, *n* (%)	421 (44.1%)	120 (39.0%)	0.11
Body mass index, mean ± SD	26.2 (4.8)	25.3 (5.2)	0.07
Laboratory values			
∙ Albumin, mean ± SD	39.7 (4.2)	38.7 (4.7)	0.02
∙ eGFR, mean ± SD	71.5 (25.0)	69.0 (24.6)	0.93
Postural hypotension, *n* (%)	395 (41.7%)	121 (42.2%)	0.89
Walking aid, *n* (%)	535 (55.0%)	9 (60.0%)	0.70
Polypharmacy, *n* (%)	674 (70.1%)	244 (72.8%)	0.35
Impaired cognition, *n* (%)	295 (39.9%)	127 (52.3%)	< 0.01
Symptoms of depression, *n* (%)	192 (19.5%)	65 (20.1%)	0.81
Katz-15, median (interquartile range)	4.0 (6.0)	3.5 (7.0)	0.40

*SD* standard deviation, *CCI* Charlson comorbidity index, *eGFR* estimated glomerular filtration rate

**Table 4:** Baseline characteristics for the different geriatric outpatients clinics

Characteristic	Referred to			*p* value
	Memory clinic	Falls clinic	Geriatric day hospital	
Age, mean ± SD	78.5 (6.7)	80.5 (6.3)	79.6 (6.6)	< 0.01
Female, *n* (%)	55.0%	68.1%	68.3%	< 0.01
eGFR				
< 30 ml/min/1.73 m^2^	3.7%	2.4%	8.9%	< 0.01
30–44 ml/min/1.73 m^2^	9.3%	13.3%	14.0%	0.10
45–59 ml/min/1.73 m^2^	20.7%	18.6%	24.1%	0.28
≥ 60 ml/min/1.73 m^2^	66.3%	65.7%	53.0%	< 0.01
CCI, mean ± SD	5.2 (2.2)	5.4 (2.2)	5.5 (2.1)	0.10
Diabetes, *n* (%)	25.1%	26.3%	27.6%	0.73
Cardiovascular disease, *n* (%)	29.1%	35.7%	35.9%	0.08
Current smoker, *n* (%)	14.2%	7.3%	13.9%	0.04
Alcohol use, *n* (%)	51.8%	41.9%	35.2%	< 0.01
Katz-15, median (IQR)	4.0 (6.0)	4.0 (5.0)	5.0 (7.0)	< 0.01
Postural hypotension, *n* (%)	39.4%	39.3%	47.2%	0.08
Walking aid, *n* (%)	42.0%	71.3%	63.2%	< 0.01
Polypharmacy, *n* (%)	62.5%	74.0%	78.0%	< 0.01
Cognitive impairment, *n* (%)	48.7%	13.8%	26.7%	< 0.01
Symptoms of depression, *n* (%)	17.3%	14.4%	26.0%	< 0.01
Accidental fall, *n* (%)	49.6%	88.6%	60.0%	< 0.01
Frequent falls, *n* (%)	30.0%	74.8%	43.1%	< 0.01

*SD* standard deviation, *IQR* interquartile range, *CCI* Charlson comorbidity index, *Accidental fall* fall in the previous year, *Frequent falls*, ≥ 2 falls in the previous year

**Table 5:** Association between a decreased eGFR and falls in patients visiting the memory clinic (*n* = 460)

eGFR-group	Model 1			Model 2			Model 3			Model 4		
	OR	CI (95%)	*p* value	OR	CI (95%)	*p* value	OR	CI (95%)	*p* value	OR	CI (95%)	*p* value
Accidental falls												
0–45 vs. > 60	1.68	0.96–2.93	0.07	1.23	0.69–2.22	0.48	0.88	0.47–1.64	0.68	0.87	0.46–1.63	0.65
45–59 vs. ≥ 60	1.44	0.91–2.28	0.12	1.13	0.70–1.84	0.62	1.05	0.63–1.73	0.86	1.02	0.61–1.70	0.94
Frequent falls												
0–45 vs. > 60	1.30	0.73–2.32	0.37	1.13	0.62–2.07	0.69	0.72	0.37–1.40	0.33	0.69	0.35–1.34	0.27
45–59 vs. ≥ 60	1.34	0.83–2.16	0.24	1.16	0.70–1.93	0.56	1.03	0.60–1.78	0.92	0.98	0.56–1.72	0.95

Model 1 unadjusted, model 2: adjusted for age and gender, model 3: adjusted for model 2 Katz-15, the use of a walking aid, polypharmacy. Model 4: adjusted for model 3 + diabetes, cardiovascular disease, alcohol, postural hypotension, cognitive impairment, symptoms of depression

*eGFR* estimated glomerular filtration rate in ml/min/1.72 m^2^, *Accidental fall* fall in the previous year, *Frequent falls* ≥ 2 falls in the previous year, *OR* odds ratio, *CI* (95%) 95% confidence interval

**Table 6:** Association between a decreased eGFR and falls in patients visiting the falls clinic (*n* = 210)

eGFR-group	Model 1			Model 2			Model 3			Model 4		
	OR	CI (95%)	*p* value	OR	CI (95%)	*p* value	OR	CI (95%)	*p* value	OR	CI (95%)	*p* value
Accidental falls												
0–45 vs. > 60	0.60	0.20–1.81	0.36	0.48	0.15–1.53	0.21	0.39	0.12–1.32	0.13	0.39	0.11–1.45	0.16
45–59 vs. ≥ 60	0.59	0.21–1.65	0.31	0.53	0.18–1.57	0.25	0.49	0.16–1.48	0.21	0.33	0.10–1.10	0.07
Frequent falls												
0–45 vs. > 60	0.89	0.37–2.18	0.80	0.87	0.35–2.16	0.76	0.85	0.33–2.18	0.74	0.79	0.30–2.12	0.64
45–59 vs. ≥ 60	0.80	0.36–1.78	0.58	0.81	0.35–1.85	0.61	0.85	0.36–1.97	0.70	0.74	0.31–1.79	0.51

Model 1 unadjusted, model 2: adjusted for age and gender, model 3: adjusted for model 2 Katz-15, the use of a walking aid, polypharmacy. Model 4: adjusted for model 3 + diabetes, cardiovascular disease, alcohol, postural hypotension, cognitive impairment, symptoms of depression

*eGFR* estimated glomerular filtration rate in ml/min/1.72 m^2^, *Accidental fall* fall in the previous year, *Frequent falls* ≥ 2 falls in the previous year, *OR* odds ratio, *CI* (95%) 95% confidence interval

**Table 7:** Association between a decreased eGFR and falls in patients visiting the geriatric assessment clinic (*n* = 315)

eGFR-group	Model 1			Model 2			Model 3			Model 4		
	OR	CI (95%)	*p* value	OR	CI (95%)	*p* value	OR	CI (95%)	*p* value	OR	CI (95%)	*p* value
Accidental falls												
0–45 vs. > 60	2.33	1.27–4.27	< 0.01	2.30	1.23–4.30	< 0.01	2.20	1.13–4.27	0.02	2.08	1.06–4.08	0.03
45–59 vs. ≥ 60	1.22	0.70–2.12	0.48	1.18	0.67–2.07	0.57	1.07	0.59–1.94	0.82	0.92	0.50–1.70	0.80
Frequent falls												
0–45 vs. > 60	1.89	1.08–3.31	0.03	1.91	1.07–3.41	0.03	1.56	0.84–2.89	0.16	1.60	0.85–2.99	0.14
45–59 vs. ≥ 60	1.51	0.87–2.62	0.14	1.48	0.85–2.61	0.17	1.29	0.72–2.31	0.39	1.18	0.64–2.17	0.60

Model 1 unadjusted, model 2: adjusted for age and gender, model 3: adjusted for model 2 KATZ-15, the use of a walking aid, polypharmacy. Model 4: adjusted for model 3 + diabetes, cardiovascular disease, alcohol, postural hypotension, cognitive impairment, symptoms of depression

*eGFR* estimated glomerular filtration rate in ml/min/1.72 m^2^, *Accidental fall* fall in the previous year, *Frequent falls* ≥ 2 falls in the previous year, *OR* odds ratio, *CI* (95%) 95% confidence interval

## References

[1] World Health Organization (2007) WHO global report on falls prevention in older age, vol 53. Community Health (Bristol) [Internet]. http://www.who.int/ageing/publications/Falls_prevention7March.pdf

[2] Masud T, Morris RO (2001). Epidemiology of falls. Age Ageing.

[3] Rubenstein LZ (2006). Falls in older people: epidemiology, risk factors and strategies for prevention. Age Ageing.

[4] Bowling CB, Bromfield SG, Colantonio LD, Gutierrez OM, Shimbo D, Reynolds K (2016). Association of reduced eGFR and albuminuria with serious fall injuries among older adults. Clin J Am Soc Nephrol.

[5] Heinrich S, Rapp K, Rissmann U, Becker C, König HH (2010). Cost of falls in old age: a systematic review. Osteoporos Int.

[6] Dutra MC, Uliano EJ, Machado DF, Martins T, Schuelter-Trevisol F, Trevisol DJ (2014). Assessment of kidney function in the elderly: a population-based study. J Bras Nefrol.

[7] Jha V, Garcia-Garcia G, Iseki K, Li Z, Naicker S, Plattner B (2013). Chronic kidney disease: global dimension and perspectives. Lancet (London).

[8] Bowling C, Booth JN, Gutierrez OM, Kurella Tamura M, Judd S, Warnock D (2014). Nondisease-specific problems and all-cause mortality among older adults with chronic kidney disease: findings from the REasons for geographic and racial differences in stroke (REGARDS) study. J Am Geriatr Soc.

[9] Heymann EP, Jenkins M, Goldsmith D (2012). Clinical features and manifestations of CKD-MBD. Clin Rev Bone Miner Metab.

[10] Abdel-Rahman EM, Turgut F, Turkmen K, Balogun RA (2011). Falls in elderly hemodialysis patients. QJM.

[11] Farragher J, Chiu E, Ulutas O, Tomlinson G, Cook WL, Jassal SV (2014). Accidental falls and risk of mortality among older adults on chronic peritoneal dialysis. Clin J Am Soc Nephrol.

[12] Lopez-Soto PJ, De Giorgi A, Senno E, Tiseo R, Ferraresi A, Canella C (2015). Renal disease and accidental falls: a review of published evidence. BMC Nephrol.

[13] Hall RK, Landerman LR, O’Hare AM, Anderson RA, Colon-Emeric CS (2015). Chronic kidney disease and recurrent falls in nursing home residents: a retrospective cohort study. Geriatr Nurs.

[14] Dukas LC, Schacht E, Mazor Z, Stähelin HB (2005). A new significant and independent risk factor for falls in elderly men and women: a low creatinine clearance of less than 65 ml/min. Osteoporos Int.

[15] Gallagher JC, Rapuri P, Smith L (2007). Falls are associated with decreased renal function and insufficient calcitriol production by the kidney. J Steroid Biochem Mol Biol.

[16] Hall RK, Erman LR, O’Hare AM, Anderson RA, Colón-Emeric CS (2015). Chronic kidney disease and recurrent falls in nursing home residents: a retrospective cohort study. Geriatr Nurs.

[17] Charlson ME, Pompei P, Ales KL, MacKenzie CR (1987). A new method of classifying prognostic comorbidity in longitudinal studies: development and validation. J Chronic Dis.

[18] Laan W, Zuithoff NPA, Drubbel I, Bleijenberg N, Numans ME, de Wit NJ (2014). Validity and reliability of the Katz-15 scale to measure unfavorable health outcomes in community-dwelling older people. J Nutr Health Aging.

[19] Folstein MF, Robins LN, Helzer JE (1983). The Mini-Mental State Examination. Arch Gen Psychiatry.

[20] Yesavage JA, Sheikh JI (1986). 9/Geriatric Depression Scale (GDS). Clin Gerontol.

[21] Levey AS, Coresh J, Bolton K, Culleton B, Harvey KS, Ikizler TA, Johnson CA, Kausz A, Kimmel PL, Kusek J, Levin A (2002). K/DOQI clinical practice guidelines for chronic kidney disease: evaluation, classification, and stratification. Am J Kidney Dis.

[22] Deandrea S, Lucenteforte E, Bravi F, Foschi R, La Vecchia C, Negri E (2010). Risk factors for falls in community-dwelling older people. Epidemiology.

[23] Soriano T, DeCherrie LV, Thomas DC (2007). Falls in the community-dwelling older adult: a review for primary-care providers. Clin Interv Aging.

[24] Polinder-Bos HA, Emmelot-Vonk MH, Gansevoort RT, Diepenbroek A, Gaillard CAJM (2014). High fall incidence and fracture rate in elderly dialysis patients. Neth J Med.

[25] Jansen S, Bhangu J, de Rooij S, Daams J, Kenny RA, van der Velde N (2016). The association of cardiovascular disorders and falls: a systematic review. J Am Med Dir Assoc.

[26] Sedaghat S, Darweesh SKL, Verlinden VJA, van der Geest JN, Dehghan A, Franco OH (2018). Kidney function, gait pattern and fall in the general population: a cohort study. Nephrol Dial Transplant.

[27] Odden MC, Chertow GM, Fried LF, Newman AB, Connelly S, Angleman S (2006). Cystatin C and measures of physical function in elderly adults: the Health, Aging, and Body Composition (HABC) Study. Am J Epidemiol.

[28] König M, Gollasch M, Spira D, Buchmann N, Hopfenmüller W, Steinhagen-Thiessen E (2018). Mild-to-moderate chronic kidney disease and geriatric outcomes: analysis of cross-sectional data from the Berlin Aging Study II. Gerontology.

[29] Cummings SR, Nevitt MC, Kidd S (1988). Forgetting falls. The limited accuracy of recall of falls in the elderly. J Am Geriatr Soc.

[30] Stevens J, Ballesteros MF, Mack K, Rudd R, DeCaro E, Adler G (2012). Gender differences in seeking care for falls in the aged medicare population. Am J Prev Med.

[31] Nussbaum J (2013). Fall prevention, reduced morbidity, and improved functional outcome measures in frail patients with end stage kidney disease undergoing a skilled physical therapy program: the prohealth experience. Blood Purif.

[32] Chang JT, Morton SC, Rubenstein LZ, Mojica WA, Maglione M, Suttorp MJ (2004). Interventions for the prevention of falls in older adults: systematic review and meta-analysis of randomised clinical trials. Br Med J.

[33] Heung M, Adamowski T, Segal JH, Malani PN (2010). A successful approach to fall prevention in an outpatient hemodialysis center. Clin J Am Soc Nephrol.

